# Lipid Accumulation Product Combined With Urine Glucose Excretion Improves the Efficiency of Diabetes Screening in Chinese Adults

**DOI:** 10.3389/fendo.2021.691849

**Published:** 2021-08-23

**Authors:** Juan Chen, Hong Sun, Shanhu Qiu, Hu Tao, Jiangyi Yu, Zilin Sun

**Affiliations:** ^1^Department of Endocrinology, Jiangsu Province Hospital of Chinese Medicine, Affiliated Hospital of Nanjing University of Chinese Medicine, Nanjing, China; ^2^Department of Endocrinology, Zhongda Hospital, Institute of Diabetes, Medical School, Southeast University, Nanjing, China; ^3^Department of Endocrinology and Metabolism, The First Affiliated Hospital of Soochow University, Suzhou, China; ^4^Department of Endocrinology, Shenzhen People’s Hospital, The Second Clinical Medical College of Jinan University, The First Affiliated Hospital of Southern University of Science and Technology, Shenzhen, China; ^5^School of Mechanical Engineering, and Jiangsu Key Laboratory for Design and Manufacture of Micro-Nano Biomedical Instruments, Southeast University, Nanjing, China

**Keywords:** lipid accumulation product, urine glucose excretion, newly diagnosed diabetes, prediabetes, diabetes screening

## Abstract

**Background:**

To compare the efficacy of lipid accumulation product (LAP) and urine glucose excretion (UGE) in predicting diabetes and evaluate whether the combination of LAP and UGE would help to improve the efficacy of using LAP alone or UGE alone in identifying diabetes.

**Methods:**

Data from 7485 individuals without prior history of diabetes who participated in a cross-sectional survey in Jiangsu, China, were analyzed. Each participant underwent an oral glucose-tolerance test. Operating characteristic curves (ROC) and logistic regression analyses were used to evaluate the performance of LAP and UGE in identification of newly diagnosed diabetes (NDM) and prediabetes (PDM).

**Results:**

For subjects with NDM, the area under the ROC curve was 0.72 for LAP and 0.85 for UGE, whereas for PDM, these values were 0.62 and 0.61, respectively. Furthermore, LAP exhibited a comparable sensitivity with UGE in detecting NDM (76.4% *vs* 76.2%, p = 0.31). In predicting PDM, LAP showed a higher sensitivity than UGE (66.4% *vs* 42.8%, p < 0.05). The combination of LAP and UGE demonstrated a significantly higher sensitivity than that of LAP alone and UGE alone for identification of NDM (93.6%) and PDM (80.1%). Moreover, individuals with both high LAP and high UGE had significantly increased risk of NDM and PDM than those with both low LAP and low UGE.

**Conclusions:**

The combination of LAP and UGE substantially improved the efficacy of using LAP and using UGE alone in detecting diabetes, and may be a novel approach for mass screening in the general population.

## Introduction

There are evidences that a variety of physiological aberrations caused by obesity mainly depend on the distribution of body fat, instead of overweight or obesity per se (1, 2). Of note, it has been reported that visceral fat, but not subcutaneous fat, is strongly associated with metabolic risk factors (3). Computed tomography (CT) or magnetic resonance imaging is considered as the gold standard for assessing lipid accumulation and distribution patterns. Yet they cannot be widely applied in clinical practice since they are costly and time-consuming. In addition, CT examination is radioactive. Lipid accumulation product (LAP), based on a combination of waist circumference (WC) and triglyceride (TG), has been recognized as a novel marker of visceral adipose accumulation (4). The efficacy of LAP for evaluating visceral fat has been demonstrated (5). In addition to the simple calculation, LAP does not require high expenditure of time and cost, thus making it suitable for a large sample population. According to prior research, accumulation of visceral fat measured by CT over 5 years is independently associated with greater risk of incident diabetes (6). Furthermore, several studies have suggested that LAP may serve as a useful predictor of insulin resistance (4, 7). Collectively, LAP may be a useful marker to predict diabetes. However, LAP was derived from studies of the US civilian, noninstitutionalized population that included an oversample of non-Hispanic blacks and Mexican Americans. The studies focused on LAP in Chinese population with large sample size are relatively scare.

Due to increased glucose reabsorption, which has been confirmed in individuals with diabetes, the role of kidney in glucose homeostasis has become a research focus in recent years (8). Currently, promoting urine glucose excretion (UGE) through inhibition of renal glucose reabsorption has been demonstrated to be an effective strategy for the treatment of diabetes (9). In addition, UGE reliably reflects the prevailing plasma glucose levels (10, 11). In our previous work, we demonstrated that UGE may be a practical and reliable approach for mass screening for diabetes, especially in developing countries (12). Moreover, urine glucose has also been confirmed as the strongest predictor of gestational diabetes due to its noninvasiveness and availability to pregnant women (13). Recently, UGE has gained much more attention because of its significance in clinical practice, such as diabetes screening and glycemic control (12, 14). However, its utility for predicting pre-diabetes (PDM), a high-risk state for diabetes, is limited. We found that the sensitivity of UGE for the estimation of 2h-PG ≥ 7.8 mmol/L was 52.3%, suggesting that nearly half of PDM cases could not be detected by using UGE alone (15). So, we attempted to improve the efficacy of UGE in diabetes screening by introducing new strategies.

Therefore, in the present study, we aimed to confirm the efficacy of LAP in identifying diabetes in Chinese population and compare the efficacy of LAP and UGE in predicting diabetes, and further evaluate whether the combination of LAP and UGE would help to improve the efficacy of using LAP alone or UGE alone in identifying diabetes.

## Materials and Methods

### Study Design and Participants

Data were derived from a cross-sectional survey carried out in six cities in Jiangsu Province, China, between November 12, 2015 and June 28, 2016 (15, 16). Individuals aged between 18 and 65 years old, and without prior history of diabetes or taking anti-diabetic medication were invited to take part in this study. This study was conducted in accordance with the 1975 Declaration of Helsinki and was approved by the ethical review committee of Jiangsu Provincial Center for Disease Control and Prevention (JSJK2016 B003 03). Each participant provided written informed consent. A total of 7689 Chinese people participated in the survey. Among them, 204 subjects were further excluded, because they missed the measurement of glycated hemoglobin A1c (HbA1c) or UGE. Therefore, 7485 subjects were included in the final analyses (16).

### Anthropometric and Laboratory Measurements

All eligible participants were interviewed using a structured questionnaire to obtain information on demographic characteristics and medical histories. Heart rate (HR), blood pressure (BP), WC, height, and weight were measured with standardized protocols, as previous reported (17, 18). Each participant was invited to take an oral glucose tolerance test (OGTT). HbA1c, fasting plasma glucose (FPG), 2 h plasma glucose (2h-PG), TG, total cholesterol (TC), blood urea nitrogen (BUN), and creatinine were measured. Estimation of glomerular filtration rate (eGFR) was calculated using the CKD-EPI equation based on creatinine level (19). The participants were asked to empty their bladders before they were given an OGTT. All the urine samples were collected over a 2 h period after oral glucose loading. During this period, the participants were not allowed to drink water or undertake strenuous exercise. UGE was calculated as the urine glucose concentration (mg/dl) × urine volume (dl), as previously described (16). According to previous research, LAP was calculated as LAP = (WC−65) × TG for men and LAP = (WC−58) × TG for women (4).

### Definitions

Based on the 2012 American Diabetes Association (ADA) criteria, newly diagnosed diabetes (NDM) was defined as the absence of previous diagnosis or treatment for diabetes, and FPG ≥ 7.0 mmol/L and/or 2h-PG ≥ 11.1 mmol/L and/or HbA1c ≥ 6.5%. PDM was defined as FPG ≥ 5.6 mmol/L and ≤ 6.9 mmol/L, 2h PG ≥ 7.8 mmol/L and < 11.0 mmol/L, or 5.7%≤ HbA1c ≤ 6.4%. LAP exceeding the optimal cutoff point determined in the present study was considered as high LAP (H-LAP), while LAP less than the optimal cutoff point was considered as low LAP (L-LAP). UGE greater than the optimal cutoff point determined in this study was defined as high UGE (H-UGE), otherwise it was considered as low UGE (L-UGE).

### Statistical Analysis

Assuming a sensitivity of 76%, a specificity of 89%, and a disease prevalence of 10%, the allowable error was to 0.1 with α 0.05, at least 700 subjects were required (12). Continuous variables were presented as means ± SD, or median (25th to 75th percentiles). Categorical variables were presented as numbers (percentages). The characteristics of the participants in the different groups were compared using one-way analysis of variance (ANOVA) for continuous variables and χ2 test for categorical variables. Non-parametric Kruskal-Wallis test followed by all pairwise multiple comparisons was performed when the data distribution was skewed. The associations of LAP and UGE with other clinical indicators were examined using Spearman’s correlation. Receiver operating characteristic (ROC) curves were generated and the areas under the curves (AUC) were used to evaluate the efficacy of LAP and UGE in detecting NDM and PDM. The sensitivity and specificity were calculated. The optimal cutoff point was determined using the maximum of Youden’s index. Logistic regression analyses with adjustment of age, genders, BP, TC, body mass index (BMI), BUN, and creatinine as the confounding factors, were performed to obtain the odds ratios (OR) for NDM and PDM among subjects with H-LAP and H-UGE that was defined according to the cutoff points determined in this study. A p value < 0.05 was considered statistically significant. All statistical analyses were conducted using SPSS 22.0 (SPSS Inc, Chicago, IL, USA).

## Results

### General Characteristics of the Study Participants

The general characteristics of the study population, according to glucose tolerance status, are presented in **Table 1**. Individuals with NDM and PDM were older, had higher BP, FPG, 2h-PG, TC, TG, and showed a greater BMI, compared with normal glucose tolerance (NGT) subjects, while there was no difference in creatinine levels. In addition, individuals with NDM and PDM showed significantly higher LAP in comparison to those with NGT. NDM subjects had highest levels of LAP. Moreover, NDM subjects also exhibited significantly higher UGE than PDM and NGT subjects.

**Table 1:** Characteristics of the study participants according to glucose tolerance status.

	Total (n = 7485)	NGT (n = 3243)	PDM (n = 3645)	NDM (n = 597)
**Age (years)**	43.9 ± 11.9	39.2 ± 11.5	47.0 ± 11.1^*^	50.0 ± 9.8^*^ ^†^
**Male (%)**	3298 (44.1%)	1362 (42.0%)	1644 (45.1%)^*^	292 (48.9%)^*^
**HR (beats/min)**	77.6 ± 13.3	77.6 ± 15.3	77.3 ± 11.5	79.4 ± 12.2^*^ ^†^
**Blood pressure (mmHg)**				
** Systolic**	128.3 ± 19.0	123.3 ± 17.7	131.0 ± 18.9^*^	139.6 ± 18.4^*^ ^†^
** Diastolic**	79.2 ± 14.5	77.0 ± 17.0	80.3 ± 11.9^*^	84.4 ± 11.3^*^ ^†^
**Plasma glucose (mmol/L)**				
** FPG**	5.5 ± 1.0	5.0 ± 0.3	5.5 ± 0.5^*^	7.5 ± 2.1^*^ ^†^
** 2h-PG**	6.6 ± 2.6	5.5 ± 1.0	6.6 ± 1.6^*^	12.5 ± 4.4^*^ ^†^
**HbA1c (%)**	5.7 ± 0.6	5.3 ± 0.2	5.8 ± 0.3^*^	7.0 ± 1.3^*^ ^†^
**TC (mmol /L)**	4.7 ± 0.9	4.5 ± 0.9	4.8 ± 0.9^*^	5.1 ± 1.0^*^ ^†^
**TG (mmol /L)**	1.2 (0.9 - 1.8)	1.1 (0.8 - 1.6)	1.3 (0.9 - 1.9)^*^	1.7 (1.2 - 2.5)^*^ ^†^
**BUN (mmol /L)**	5.0 ± 1.5	4.8 ± 1.4	5.2 ± 1.6^*^	5.2 ± 1.5^*^
**Creatinine (umol /L)**	74.0 (62.0-83.8)	74.2 (62.8 - 84.0)	73.6 (62.0 - 83.2)	73.3 (59.8 - 85.0)
**eGFR (ml/min/1.73 m^2^)**	96.8 ± 17.5	99.2 ± 17.3	95.0 ± 17.4^*^	94.4 ± 17.3^*^
**BMI**	25.2 ± 4.0	24.1 ± 3.8	25.7 ± 3.9^*^	27.4 ± 3.9^*^ ^†^
**LAP**	26.9 (14.3 - 48.3)	20.8 (11.2 - 38.0)	30.0 (16.9 - 51.3)^*^	49.9 (30.3 - 77.9)^*^ ^†^
**UGE (mg)**	28.0 (10.0-85.0)	20.0 (6.3 - 48.0)	31.5 (11.0 - 96.0)^*^	750.0 (138.0-1975.0)^*†^

Data are presented as n (%), mean ± SD, or median (25th to 75th percentiles) as appropriate. *P < 0.05 for the difference between the indexed category and NGT, ^†^P < 0.05 for the difference between the indexed category and PDM. HR, heart rate; HbA1c, glycated hemoglobin A1c; FPG, fasting plasma glucose; 2h-PG, 2h-plasma glucose; TC, total cholesterol; TG, triglycerides; BUN, blood urea nitrogen; eGFR, estimation of glomerular filtration rate; BMI, body mass index; LAP, lipid accumulation product; UGE, urine glucose excretion; NGT, normal glucose tolerance; PDM, prediabetes; NDM, newly diagnosed diabetes.

### Correlations of LAP and UGE With Glycemic Variables

LAP was significantly associated with FPG (r = 0.221, p < 0.01), 2h-PG (r = 0.391, p < 0.01), and HbA1c (r = 0.243, p < 0.01) in the overall population. Furthermore, UGE was also positively correlated with FPG (r = 0.342, p < 0.01), 2h-PG (r = 0.271, p < 0.01), and HbA1c (r = 0.253, p < 0.01).

### Performance of LAP and UGE in Predicting NDM and PDM

**Figure 1:**
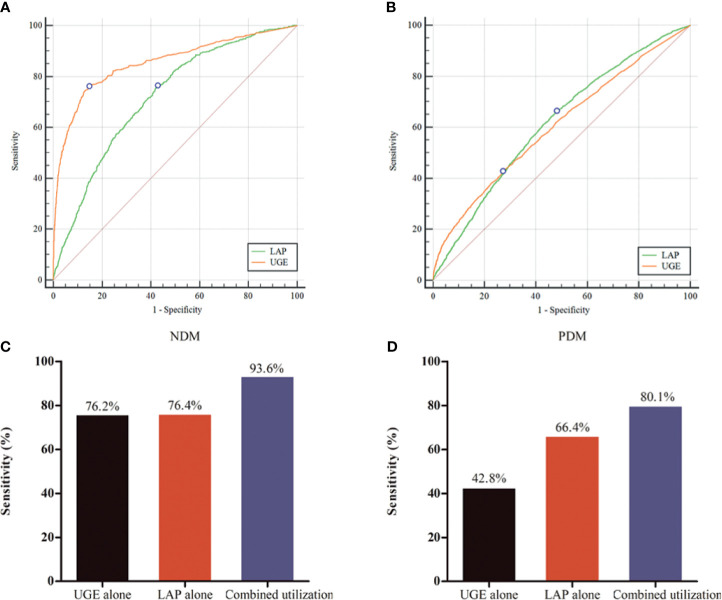
Performance of lipid accumulation product (LAP) and urine glucose excretion (UGE) in predicting for newly diagnosed diabetes (NDM) and prediabetes (PDM). **(A)** Receiver operating characteristic (ROC) curve for identifying NDM. **(B)** ROC curve for identifying PDM. **(C)** Evaluation of LAP combined with UGE for identifying NDM. **(D)** Evaluation of LAP combined with UGE for identifying PDM.

ROC curves represent the diagnostic accuracy of LAP and UGE for the estimation of NDM and PDM. The AUC for LAP was significantly lower than that for UGE for predicting NDM (P < 0.001), with AUC values of 0.72 (95%CI: 0.71 - 0.73), 0.85 (95%CI: 0.85 - 0.86), respectively (**Figure 1A**). However, LAP showed a comparable sensitivity with UGE in detecting NDM (P = 0.31). LAP displayed a sensitivity of 76.4% and a specificity of 57.3% at the optimal cutoff point of 29.6 for the prediction of NDM. Additionally, UGE exhibited a sensitivity of 76.2% and a specificity of 85.4% for the detection of NDM at a corresponding optimal cutoff point of 130mg (**Table 2**).

**Table 2:** Performance of LAP and UGE for predicting NDM and PDM.

	AUC	Optimal cutoff point	Sensitivity (%)	Specificity (%)
**For NDM**				
**LAP**	0.72 (0.71 - 0.73)	29.6	76.4 (72.8 - 79.7)	57.3 (56.1 - 58.5)
**UGE**	0.85 (0.85 - 0.86)	130.0	76.2 (72.6 - 79.6)	85.4 (84.5 - 86.2)
**For PDM**				
**LAP**	0.62 (0.60 - 0.63)	21.6	66.4 (64.8 - 67.9)	52.0 (50.2 – 53.7)
**UGE**	0.61 (0.59 - 0.62)	42.0	42.8 (41.2 - 44.5)	72.9 (71.3 - 74.4)

Data are means (95% confidence interval). NDM, newly diagnosed diabetes; PDM, pre-diabetes; LAP, lipid accumulation product; UGE, urine glucose excretion; AUC, the area under the ROC curves.

For identifying PDM, the AUC was 0.62 for LAP and 0.61 for UGE (**Figure 1B**). The optimal LAP cutoff value in this study population was 21.6 and the corresponding sensitivity and specificity were 66.4% and 52.0%, respectively (**Table 2**). Moreover, at the optimal UGE cutoff point of 42mg for PDM, the sensitivity and specificity were 42.8% and 72.9%, respectively. LAP (66.4%, 95%CI: 64.8% - 67.9%) showed a significantly higher sensitivity over UGE (42.8%, 95%CI: 41.2% - 44.5%) in detecting PDM (p < 0.05).

### Evaluation of LAP Combined With UGE in Predicting Diabetes

As shown in **Figures 1C, D**, the combined utilization of LAP and UGE showed a significantly higher sensitivity than that of LAP alone and UGE alone for the identification of NDM (both P < 0.001). Further analysis showed that combined utilization of LAP and UGE had an absolute sensitivity advantage of 22.5% over LAP alone and 22.8% over UGE alone. In addition, the sensitivity of using UGE alone to identify PDM was 42.8%, while the combined utilization of LAP and UGE was 80.1%, showing an absolute sensitivity advantage of 87.1% over UGE alone. Besides, the combined utilization of LAP and UGE had an absolute sensitivity advantage of 20.6% over LAP alone.

### Logistic Regression Analyses of Odds Ratios for NDM and PDM

**Figure 2:**
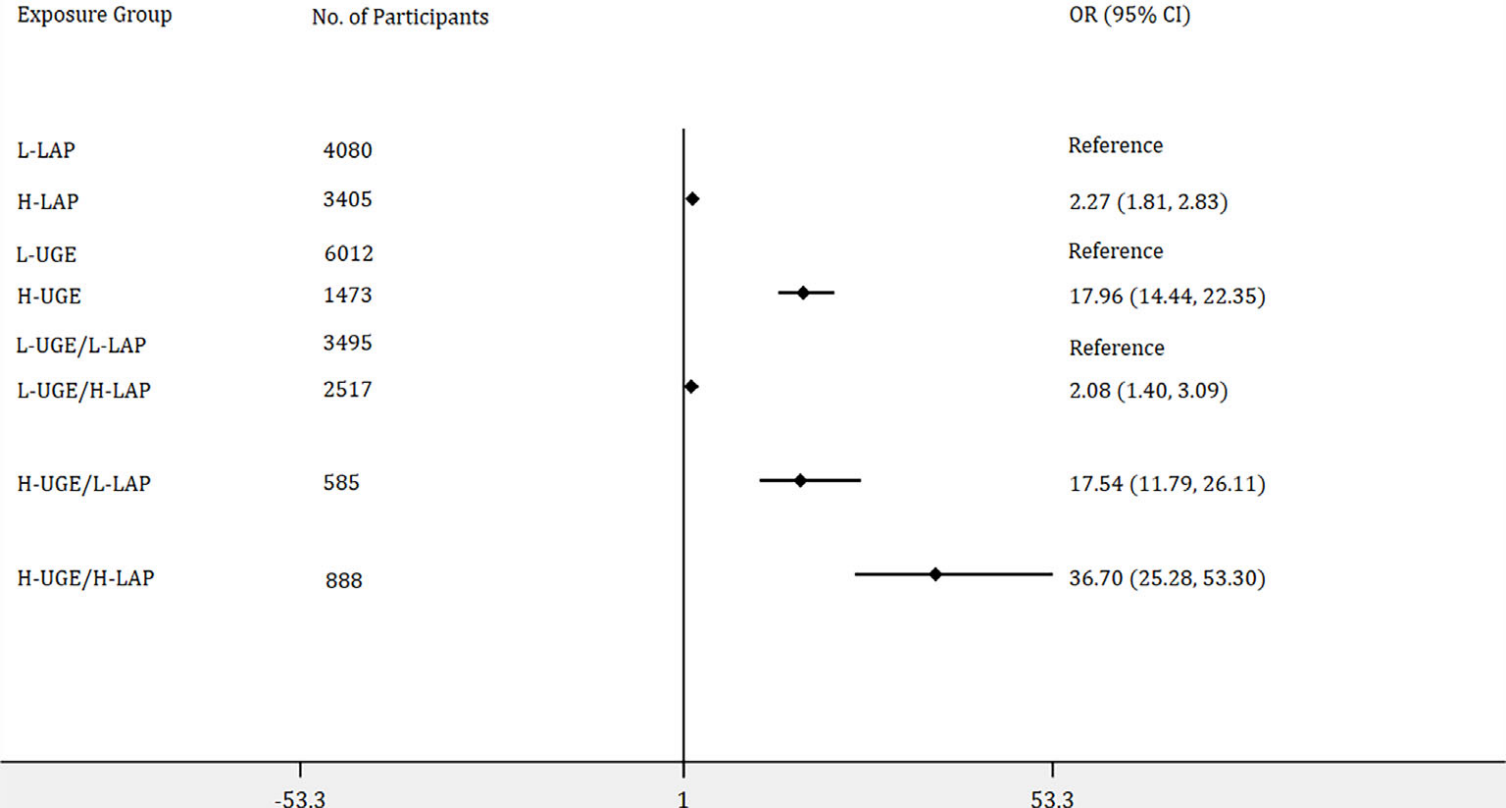
Logistic regression analyses of odds ratios for newly diagnosed diabetes (NDM) with adjustment of age, genders, blood pressure, total cholesterol, body mass index, blood urea nitrogen, and creatinine. The association of lipid accumulation product (LAP) with the risk of NDM was assessed by dividing the participants into two groups: (1) low LAP (L-LAP), (2) high LAP (H-LAP). The L-LAP group was used as a reference in the analysis. The association of urine glucose excretion (UGE) with the risk of NDM was assessed by dividing the participants into low UGE (L-UGE) group and high UGE (H-UGE) group. The L-UGE group was used as a reference in the analysis. In addition, the joint association of UGE and LAP was assessed by dividing the participants into four groups: (1) L-UGE/L-LAP, (2) L-UGE/H-LAP, (3) H-UGE/L-LAP, (4) H-UGE/H-LAP. The L-UGE/L-LAP group was used as a reference in the analysis. The forest plot was displayed in odds ratio and 95% confidence interval.

Furthermore, binary logistic regression analyses were performed to identify the associations of LAP and UGE with the risk of NDM and PDM. In addition, the joint association of LAP and UGE with the risk of NDM and PDM was assessed by dividing the participants into four groups: L-UGE/L-LAP, L-UGE/H-LAP, H-UGE/L-LAP, and H-UGE/H-LAP. As shown in **Figure 2**, among the total population, 3405 (45.5%) participants exhibited H-LAP and 1473 (19.7%) showed H-UGE. Subjects with H-LAP were more likely to have NDM (OR = 2.27, 95% CI: 1.81- 2.83, p < 0.001). Moreover, subjects with H-UGE showed a 17.96-fold increased odds ratio for NDM compared with those with L-UGE. In addition, 3495 (46.7%) subjects showed both L-LAP and L-UGE, while 888 (11.9%) displayed both H-LAP and H-UGE. Further analysis showed that individuals with H-UGE/H-LAP displayed higher risk of NDM (OR = 36.70, 95% CI: 25.28 - 53.30) than those with L-UGE/H-LAP (OR = 2.08, 95% CI: 1.40 - 3.09) and H-UGE/L-LAP (OR = 17.54, 95% CI: 11.79 - 26.11).

**Figure 3:**
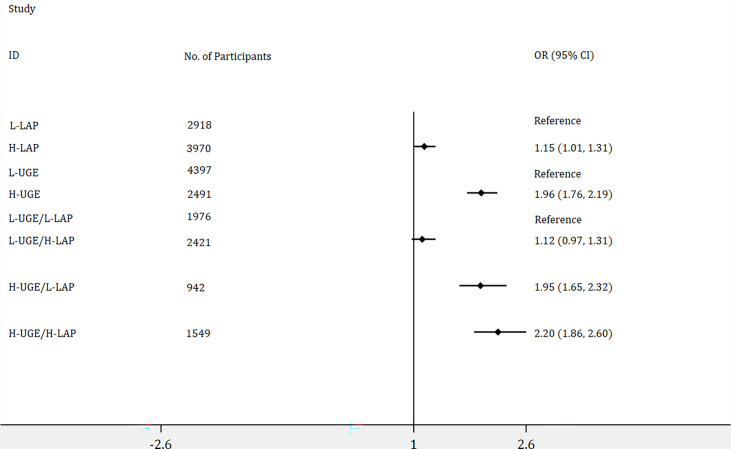
Logistic regression analyses of odds ratios for prediabetes (PDM) with adjustment of age, genders, blood pressure, total cholesterol, body mass index, blood urea nitrogen, and creatinine. The association of lipid accumulation product (LAP) with the risk of PDM was assessed by dividing the participants into two groups: (1) low LAP (L-LAP), (2) high LAP (H-LAP). The L-LAP group was used as a reference in the analysis. The association of urine glucose excretion (UGE) with the risk of PDM was assessed by dividing the participants into low UGE (L-UGE) group and high UGE (H-UGE) group. The L-UGE group was used as a reference in the analysis. The joint association of UGE and LAP was assessed by dividing the participants into four groups: (1) L-UGE/L-LAP, (2) L-UGE/H-LAP, (3) H-UGE/L-LAP, (4) H-UGE/H-LAP. The L-UGE/L-LAP group was used as a reference in the analysis. The forest plot was displayed in odds ratio and 95% confidence interval.

As shown in **Figure 3**, 6888 subjects were identified as non-diabetes, of those, 3970 participants (57.6%) showed H-LAP and 2491 (36.2%) exhibited H-UGE. H-LAP and H-UGE were significantly associated with increased odds ratios for PDM. In addition, participants with H-UGE/H-LAP displayed the highest risk for PDM (OR = 2.20, 95% CI: 1.86 - 2.60, p < 0.001).

## Discussion

In this study of Chinese participants without previous history of diabetes, we found that UGE showed a better performance in discriminating NDM compared with LAP since the AUC for LAP was significantly lower than that for UGE for detecting NDM (P < 0.001). However, LAP exhibited a comparable sensitivity with UGE in detecting NDM (76.4% *vs* 76.2%, p = 0.31). In addition, in predicting PDM, LAP showed a significantly higher sensitivity than UGE (66.4% *vs* 42.8%, p < 0.05). Furthermore, the combined utilization of LAP and UGE improved the sensitivity to 93.6% for identifying NDM and to 80.1% for identifying PDM. Since LAP is easy to access and UGE is available, the combined utilization of LAP and UGE might be a practical and sufficient test model for diabetes screening.

The global rise in diabetes has become a public health crisis (20). Notably, accumulating evidences have noted that diabetes-associated complications are sometimes already present in individuals with NDM (21, 22). In addition to health burden, diabetes also leads to a huge economic burden for society (20). Due to its large population, China may bear a higher diabetes-related burden than any other countries (23). However, according to a prior research, up to 70% of the people with diabetes are left undiagnosed in China (24). More efficient but inexpensive approaches for predicting diabetes are urgently needed to improve health care for patients with diabetes.

LAP, an index describing the overaccumulation of lipid, is becoming a valuable health screening tool (25). However, LAP was derived from studies of the US civilian, noninstitutionalized population that included an oversample of non-Hispanic blacks and Mexican Americans. The studies that have been designed to focus on the screening performance of this tool in Chinese participants are relatively scare. Our work found that LAP exhibited a sensitivity of 76.4% for the detection of NDM, indicating that almost 24% subjects will miss further testing and diagnosis. Besides, a relatively lower sensitivity of 66.4% for the diagnosis of PDM was observed. However, another study indicated that LAP displayed a sensitivity of 72.3% for men at the optimal cutoff point of 8.07, and a sensitivity of 75.1% for women at the optimal cutoff point of 12.41, for diagnosis PDM (26). Moreover, our current work indicated that H-LAP was significantly associated with an increased odds ratio of NDM, as well as PDM, which is consistent with recent studies showing that LAP is strongly associated with the risk of type 2 diabetes (4, 27). Together, LAP may be a useful clinical marker for the evaluation of hyperglycemia, but the sensitivity can be further improved by introducing new strategies.

It is well known that glycosuria will appear when glycemic excursions exceed the renal threshold for glucose resorption (8). Additionally, based on the findings that UGE reflects the prevailing plasma glucose level and postprandial urine test for glucose is an effective approach for diabetes self-monitoring and self-management (28, 29). UGE could be an attractive alternative for identifying hyperglycemia. In the present study, we found positive associations between UGE and glycemic variables, including FPG, 2h-PG, and HbA1c, indicating UGE may be a useful marker for hyperglycemia. Further analyses showed that UGE exhibited a sensitivity of 76.2% for the detection of NDM, whereas a decreased sensitivity of 42.8% for the prediction of PDM. Almost 24% subjects will miss further testing for NDM and almost 58% subjects will miss further testing for PDM. The efficacy of UGE in PDM screening is limited, in consistent with a previous study (15). However, since UGE is easy to access and inexpensive (12), it should not be completely given up, especially in low−income regions.

We further investigated the sensitivity of LAP in combination with UGE, and a sensitivity of 96.3% for NDM and 80.1% for PDM were observed. Our previous wok has demonstrated that determining UGE over a specific period of time is useful for diabetes screening (17). However, the utility of UGE in PDM prediction is limited. Although oral glucose tolerance test (OGTT) is the gold standard for the diagnosis of diabetes, it is impractical to conduct the OGTT for everyone in mass screening due to its complexity (30). Notably, the efficacy of FPG for diabetes screening has been questioned. Since postprandial hyperglycemia is significantly prominent among Chinese patients with diabetes, FPG alone is not sensitive enough (15, 31). HbA1c is easy to measure. However, it is also impractical to get all individuals to use HbA1c because of the relatively high cost, especially in developing countries. In addition, the lack of standardization in HbA1c assay also limits its use for screening in Chinese population. In the present study, we found that the combination of LAP and UGE substantially improved the efficacy of using LAP alone and using UGE alone for the prediction of diabetes. Since LAP is easy to access and UGE is available, this combination may be a novel approach for mass screening in the general population.

This study was performed in a large Chinese population with no prior history of diabetes. The large sample size provided good power for data analyses. However, the limitations of our study should be considered. First, our population comprised individuals aged between 18 and 65 years old. One might speculate that the impact of LAP and UGE on diabetes diagnosis would have been even stronger in an older population. Thus, generalization of our results to older age groups should be made with caution. Second, our study only involved Chinese Han ethnic subjects, it remains largely unknown whether involving different ethnicities might contribute to differences in sensitivities. Furthermore, in the present study, 66 subjects showed renal function impairment according to the eGFR. The efficacy of UGE for identifying diabetes did not significantly improved after excluding a small number of subjects (data not shown). However, since UGE is mainly mediated by the kidneys, this approach may therefore be inappropriate for those with renal function impairment.

In conclusion, both H-LAP and H-UGE were associated with increased risk for NDM and PDM. Moreover, the combination of LAP and UGE substantially improved the efficacy of using LAP alone and using UGE alone in detecting diabetes. Due to its effectiveness, the combined utilization of UGE and LAP may be a novel approach for mass screening in the general population.

## Data Availability Statement

The raw data supporting the conclusions of this article will be made available by the authors, without undue reservation.

## Ethics Statement

The studies involving human participants were reviewed and approved by The ethical review committee of Jiangsu Provincial Center for Disease Control and Prevention. The patients/participants provided their written informed consent to participate in this study.

## Author Contributions

ZS and JY had full access to all of the data in the study and take responsibility for the integrity of the data and the accuracy of the data analysis. JC and HS contributed to the study concept and design, JC, HS, SQ, and TH contributed to the acquisition, analysis and interpretation of the data, JC drafted the manuscript. JC, HS, SQ, TH, ZS, and JY critically revised the manuscript, JC performed the statistical analyses. All authors contributed to the article and approved the submitted version.

## Funding

This study was supported by grants from National Key R&D Program of China (2016YFC1305700), National Key Scientific Instrument and Equipment Development Project of China (No. 51627808), China Postdoctoral Science Foundation (2020M671559), National Natural Science Youth Foundation of China (81700632), Natural Science Youth Foundation of Jiangsu Province (BK20170366).

## Conflict of Interest

The authors declare that the research was conducted in the absence of any commercial or financial relationships that could be construed as a potential conflict of interest.

## Publisher’s Note

All claims expressed in this article are solely those of the authors and do not necessarily represent those of their affiliated organizations, or those of the publisher, the editors and the reviewers. Any product that may be evaluated in this article, or claim that may be made by its manufacturer, is not guaranteed or endorsed by the publisher.

## References

[1] Canoy D Boekholdt SM Wareham N Luben R Welch A Bingham. Body Fat Distribution and Risk of Coronary Heart Disease in Men and Women in the European Prospective Investigation Into Cancer and Nutrition in Norfolk Cohort: A Population-Based Prospective Study. Circulation (2007) 116:2933–43. 10.1161/CIRCULATIONAHA.106.673756 18071080

[2] Després JP Lemieux I. Abdominal Obesity and Metabolic Syndrome. Nature (2006) 444:881–87. 10.1038/nature05488 17167477

[3] Ibrahim MM. Subcutaneous and Visceral Adipose Tissue: Structural and Functional Differences. Obes Rev (2010) 11:11–8. 10.1111/j.1467-789X.2009.00623.x 19656312

[4] Brahimaj A Rivadeneira F Muka T Sijbrands E Franco OH Dehghan A. Novel Metabolic Indices and Incident Type 2 Diabetes Among Women and Men: The Rotterdam Study. Diabetologia (2019) 62:1581–90. 10.1007/s00125-019-4921-2 PMC6677703 31183505

[5] Tellechea ML Aranguren F Martínez-Larrad MT Serrano-Ríos M Taverna MJ Frechtel GD. Ability of Lipid Accumulation Product to Identify Metabolic Syndrome in Healthy Men From Buenos Aires. Diabetes Care (2009) 32:e85. 10.2337/dc08-2284 19564464

[6] Wander PL Boyko EJ Leonetti DL McNeely MJ Kahn SE Fujimoto WY. Change in Visceral Adiposity Independently Predicts a Greater Risk of Developing Type 2 Diabetes Over 10 Years in Japanese Americans. Diabetes Care (2013) 36:289–93. 10.2337/dc12-0198 PMC3554282 22966093

[7] Du T Yuan G Zhang M Zhou X Sun X Yu X. Clinical Usefulness of Lipid Ratios, Visceral Adiposity Indicators, and the Triglycerides and Glucose Index as Risk Markers of Insulin Resistance. Cardiovasc Diabetol (2014) 13:146. 10.1186/s12933-014-0146-3 25326814 PMC4209231

[8] DeFronzo RA Hompesch M Kasichayanula S Liu X Hong Y Pfister M. Characterization of Renal Glucose Reabsorption in Response to Dapagliflozin in Healthy Subjects and Subjects With Type 2 Diabetes. Diabetes Care (2013) 36:3169–76. 10.2337/dc13-0387 PMC3781504 23735727

[9] Ferrannini E. Sodium-Glucose Co-Transporters and Their Inhibition: Clinical Physiology. Cell Metab (2017) 26:27–38. 10.1016/j.cmet.2017.04.011 28506519

[10] Miyashita M Ito N Ikeda S Murayama T Oguma K Kimura J. Development of Urine Glucose Meter Based on Micro-Planer Amperometric Biosensor and Its Clinical Application for Self-Monitoring of Urine Glucose. Biosens Bioelectron (2009) 24:1336–40. 10.1016/j.bios.2008.07.072 18790628

[11] Rave K Nosek L Posner J Heise T Roggen K van Hoogdalem EJ. Renal Glucose Excretion as a Function of Blood Glucose Concentration in Subjects With Type 2 Diabetes–Results of a Hyperglycaemic Glucose Clamp Study. Nephrol Dial Transplant (2006) 21:2166–71. 10.1093/ndt/gfl175 16627603

[12] Chen J Guo H Yuan S Qu C Mao T Qiu S. Efficacy of Urinary Glucose for Diabetes Screening: A Reconsideration. Acta Diabetol (2019) 56:45–53. 10.1007/s00592-018-1212-1 30159749

[13] Lawlor DA Fraser A Lindsay RS Ness A Dabelea D Catalano P. Association of Existing Diabetes, Gestational Diabetes and Glycosuria in Pregnancy With Macrosomia and Offspring Body Mass Index, Waist and Fat Mass in Later Childhood: Findings From a Prospective Pregnancy Cohort. Diabetologia (2010) 53:89–97. 10.1007/s00125-009-1560-z 19841891

[14] Lu J Bu RF Sun ZL Lu QS Jin H Wang Y. Comparable Efficacy of Self-Monitoring of Quantitative Urine Glucose With Self-Monitoring of Blood Glucose on Glycaemic Control in Non-Insulin-Treated Type 2 Diabetes. Diabetes Res Clin Pract (2011) 93(2):179–86. 10.1016/j.diabres.2011.04.012 21570146

[15] Chen J Guo HJ Qiu SH Li W Wang XH Cai M. Identification of Newly Diagnosed Diabetes and Prediabetes Using Fasting Plasma Glucose and Urinary Glucose in a Chinese Population: A Multicenter Cross-Sectional Study. Chin Med J (Engl) (2018) 131:1652–7. 10.4103/0366-6999.235884 PMC6048922 29998883

[16] Chen J Qiu S Guo H Li W Sun Z. Increased Waist-to-Hip Ratio is Associated With Decreased Urine Glucose Excretion in Adults With No History of Diabetes. Endocrine (2019) 64:239–45. 10.1007/s12020-018-1802-2 30382551

[17] Li W Xie B Qiu S Huang X Chen J Wang X. Non-Lab and Semi-Lab Algorithms for Screening Undiagnosed Diabetes: A Cross-Sectional Study. EBioMedicine (2018) 35:307–16. 10.1016/j.ebiom.2018.08.009 PMC6154869 30115607

[18] Chen Y Li W Qiu S Vladmir C Xu X Wang X. Tea Consumption and Risk of Diabetes in the Chinese Population: A Multi-Centre, Cross-Sectional Study. Br J Nutr (2020) 123:428–36. 10.1017/S000711451900299X 31760957

[19] Levey AS Stevens LA Schmid CH Zhang YL Castro AF 3rd Feldman HI. A New Equation to Estimate Glomerular Filtration Rate. Ann Intern Med (2009) 150:604–12. 10.7326/0003-4819-150-9-200905050-00006 PMC2763564 19414839

[20] Rodriguez-Gutierrez R Gonzalez-Gonzalez JG Zuñiga-Hernandez JA McCoy RG. Benefits and Harms of Intensive Glycemic Control in Patients With Type 2 Diabetes. BMJ (2019) 367:l5887. 10.1136/bmj.l5887 31690574

[21] Jing J Pan Y Zhao X Zheng H Jia Q Li H. Prognosis of Ischemic Stroke With Newly Diagnosed Diabetes Mellitus According to Hemoglobin A1c Criteria in Chinese Population. Stroke (2016) 47:2038–44. 10.1161/STROKEAHA.116.013606 27382009

[22] Zoppini G Cacciatori V Raimondo D Gemma M Trombetta M Dauriz M. Prevalence of Cardiovascular Autonomic Neuropathy in a Cohort of Patients With Newly Diagnosed Type 2 Diabetes: The Verona Newly Diagnosed Type 2 Diabetes Study (VNDS). Diabetes Care (2015) 38:1487–93. 10.2337/dc15-0081 26068862

[23] Yang W Lu J Weng J Jia W Ji L Xiao J. Prevalence of Diabetes Among Men and Women in China. N Engl J Med (2010) 362:1090–101. 10.1056/NEJMoa0908292 20335585

[24] Xu Y Wang L He J Bi Y Li M Wang T. Prevalence and Control of Diabetes in Chinese Adults. JAMA (2013) 310:948–59. 10.1001/jama.2013.168118 24002281

[25] Nascimento-Ferreira MV Rendo-Urteaga T Vilanova-Campelo RC Carvalho HB da Paz Oliveira G Paes Landim MB. The Lipid Accumulation Product Is a Powerful Tool to Predict Metabolic Syndrome in Undiagnosed Brazilian Adults. Clin Nutr (2017) 36:1693–700. 10.1016/j.clnu.2016.12.020 28081980

[26] Nusrianto R Ayundini G Kristanti M Astrella C Amalina N Muhadi. Visceral Adiposity Index and Lipid Accumulation Product as a Predictor of Type 2 Diabetes Mellitus: The Bogor Cohort Study of Non-Communicable Diseases Risk Factors. Diabetes Res Clin Pract (2019) 155:107798. 10.1016/j.diabres.2019.107798 31330161

[27] Yan G Li F Elia C Zhao Y Wang J Chen Z. Association of Lipid Accumulation Product Trajectories With 5-Year Incidence of Type 2 Diabetes in Chinese Adults: A Cohort Study. Nutr Metab (Lond) (2019) 16:72. 10.1186/s12986-019-0399-7 31641369 PMC6802349

[28] Dallosso HM Bodicoat DH Campbell M Carey ME Davies MJ Eborall HC. Self-Monitoring of Blood Glucose Versus Self-Monitoring of Urine Glucose in Adults With Newly Diagnosed Type 2 Diabetes Receiving Structured Education: A Cluster Randomized Controlled Trial. Diabetes Med (2015) 32:414–22. 10.1111/dme.12598 25308625

[29] Müller N Kämmer K Kloos C Wolf G Müller UA. Postprandial Self-Monitoring of Urine Glucose Reflects Glycaemic Control in People With Relatively Well Controlled Type 2 Diabetes Mellitus Not Treated With Insulin: A Retrospective Cohort Study. Diabetes Med (2015) 32:958–62. 10.1111/dme.12718 25659184

[30] Wang W Lee ET Fabsitz R Welty TK Howard BV. Using HbA(1c) to Improve Efficacy of the American Diabetes Association Fasting Plasma Glucose Criterion in Screening for New Type 2 Diabetes in American Indians: The Strong Heart Study. Diabetes Care (2002) 25:1365–70. 10.2337/diacare.25.8.1365 12145236

[31] Jia WP Pang C Chen L Bao YQ Lu JX Lu HJ. Epidemiological Characteristics of Diabetes Mellitus and Impaired Glucose Regulation in a Chinese Adult Population: The Shanghai Diabetes Studies, a Cross-Sectional 3-Year Follow-Up Study in Shanghai Urban Communities. Diabetologia (2007) 50:286–92. 10.1007/s00125-006-0503-1 17180353

